# What drives strategic agility? Evidence from a fuzzy-set qualitative comparative analysis (FsQCA)

**DOI:** 10.1007/s11365-022-00820-7

**Published:** 2022-12-31

**Authors:** Enrique de Diego Ruiz, Paloma Almodóvar, Ignacio Danvila del Valle

**Affiliations:** Department of Business Organisation, Economics and Business Faculty, Complutense University of Madrid, Campus de Somosaguas, Pozuelo de Alarcón (Madrid), 28223 Spain

**Keywords:** Strategic agility, Qualitative comparative analysis, Firm’s business characteristics, Firm’s business orientation, Firm’s business environment

## Abstract

Strategic agility is a topic that has not reached maturity and is of increasing interest for companies and academics alike. Yet few studies assess what drives strategic agility in organisations. This paper aims to review how companies are currently obtaining strategic agility and to identify the individual factors and configurations that lead to it. The study draws on a survey carried out with 40 Spanish companies in the services sector. The study then uses Qualitative Comparative Analysis (QCA) to identify the different configurations of factors that lead to strategic agility. Finally, we complement QCA analysis by performing a case study for each of the configurations that lead to strategic agility. The study reveals that there is no necessary condition to reach strategic agility and that companies reach it in five main ways, depending on different combinations of six factors: firm size, firm age, whether the firm is international, whether it competes in a turbulent environment, and whether the firm invests in i) capabilities and technologies, and ii) additional revenue models or cost-cutting mechanisms or not.

## Introduction

In the current global situation –where health, war and climate change challenges are intensifying the adverse effects on firms of a highly volatile, uncertain, complex and ambiguous environment– several authors have highlighted the relevance of promoting strategic agility in an attempt to foster entrepreneurial and intra-entrepreneurial responses (Ahammad et al., 2020; Gurkov & Shchetinin, 2021; Vaillant & Lafuente, 2019; Vidmar et al., 2020; Xing et al., 2020). Strategic agility is a key issue for CEOs (Doz & Kosonen, 2008a) and entrepreneurs (Kwon et al., 2018), as well as a research line that is gaining attention in the literature (de Diego & Almodovar, 2021), where several scholars have emphasised the importance of strategic agility in different industries (Appelbaum et al., 2017; Cerruti et al., 2016; Ezcan et al., 2020; Nejatian et al., 2018; Noguera et al., 2018).

However, strategic agility is a fuzzy concept, where several authors have used the term without a definition (Weill et al., 2002), while others have partially defined what it encompasses (Denning, 2018; Lewis et al., 2014; Weber & Tarba, 2014). So far, the most comprehensive definition in the literature explains that strategic agility is “a meta-capability that enables organisations to anticipate, react and seize rapid changes in the environment by redefining their corporate strategies and adapting their competitive and functional strategies to survive and create value” (de Diego & Almodovar, 2021, p. 12). Doz and Kosonen (2010) proposed that this meta-capability results from the combination of three specific capabilities: i) strategic sensitivity, ii) leadership unity, and iii) resource fluidity.

There is an increasing number of papers related to strategic agility, although there do not seem to be many studies that actually show how companies achieve it. Recent bibliometric analyses have shown how authors are writing on the topic of strategic agility across different fields (e.g., information technology, knowledge management) and industries (e.g., manufacturing) (de Diego & Almodovar, 2021), yet few studies identify which factors are related to how companies achieve strategic agility. This paper thus seeks to identify what these factors are. For this purpose, we analyse in depth 40 companies in the services sector in Spain. Using qualitative comparative analysis (QCA), we then determine which combinations of factors lead to strategic agility. QCA is a methodology that has been increasingly used by authors (Roig-Tierno et al., 2017), and which helps identify logically simplified statements that describe different combinations of conditions that indicate a specific outcome (Ragin, 2008). Use of this methodology is particularly relevant because it allows different alternatives or combinations of conditions to be found that indicate an outcome (in our case, that a company exhibits strategic agility). Additionally, it is a method which has thus far not been applied to the topic of strategic agility, such that a key contribution to the literature is therefore made.

We find that there is no 'necessary condition' to reach strategic agility and that there are five alternatives for reaching it, where different combinations of firm size, firm age, internationalisation, turbulent environments, and investments in i) technology and capabilities and ii) in revenue models and cost reduction mechanisms, are the best indicators of a company exhibiting strategic agility.

The remainder of our research is organised as follows. The next section presents the theoretical background to identify relevant factors related to strategic agility. "Methodology" describes the methodology and data collection, while “Results: obtaining the configuration/solutions and illustrations of real cases” describes the results of using the QCA tool and its connection to strategic agility. Finally, the conclusions, limitations and possible areas for future research are presented.

## Theoretical underpinnings on strategic agility

Strategic agility is a topic that has not yet reached maturity and which is studied from different perspectives (Ambituuni et al., 2021; Clauss et al., 2021; de Diego & Almodovar, 2021; Elali, 2021; Tsilionis & Wautelet, 2022). Despite being a topic that is gaining attention in the literature, there is no common definition accepted by academia (de Diego & Almodovar, 2021). However, there does seem to be a consensus that strategic agility requires three capabilities (Clauss et al., 2019; Doz, 2020; Doz & Kosonen, 2008a, b; Nejatian et al., 2019; Reed, 2021a): (a) strategic sensitivity, which refers to a capacity for proactive vigilance and awareness over changes in the environment as they develop, together with strategic firm protocols where a highly participative internal dialogue is fostered; (b) leadership unity (also labelled as collective commitment), which refers to a capability for top management to make bold joint strategic decisions quickly and accurately in response to changes in the strategic environment. This capability is derived from a collaborative and mutually dependent team with an integrative leadership style; and (c) resource fluidity, which refers to ability to reconfigure and reallocate resources and capabilities according to new strategies set out by the company, i.e., the capability to realign the structure to the business strategy.

On these grounds, we observe that strategic agility is a construct that spans several areas of the firm. It is thus conditioned by intrinsic firm characteristics and is triggered by various elements, such as a firm’s business orientation and a firm’s business environment. Among the wide range of factors that could enhance strategic agility, we identify an initiatory group of factors supported by extant theory and which are accompanied by plausible propositions (Greckhamer et al., 2018).

### Firm characteristics associated with strategic agility

Firm age has been widely studied in the literature, as it is a proxy for the experience accumulated by the firm (Almodóvar et al., 2021; Rodríguez-Ruiz et al., 2019). Several lines of research thus establish a relationship between the firm's age and different forms of business performance. Regarding this literature, several connections are observed in relation to strategic agility. For example, Thornhill and Amit (2003) conducted an analysis of 339 Canadian business bankruptcies. Among the results, they found that the failure of older firms was due to their inability to adapt to the changing environment. Delving more deeply, Loderer and Waelchli (2010) explained that firm age affected economic performance for various reasons. On the one hand, they underlined that the age of the firm generates greater organisational rigidity and, due to this greater rigidity, many resources and capabilities become obsolete, R&D investments decrease, costs increase, and business growth slows down. On the other hand, they explained that the older the firm, the lower the quality of corporate governance. This was because the size of boards of directors and CEO remuneration tended to increase. These factors led to a reduction in a firm’s problem-solving capacity. In a later work, Loderer et al. (2017) analysed listed companies between 1978 and 2013 to find out why older companies had fewer opportunities for growth. Their results pointed to the fact that, with age, firms focused more on exploitation than exploration, becoming more productive with respect to their traditional products, but less proactive in the face of a changing environment. In the same line, Coad et al. (2018) indicated that there is direct causality between age and business performance and that the effects of age might produce organisational rigidity and a firm's routinisation of protocols. All this rationale suggests that firm age has a negative effect on the different capabilities that integrate strategic agility (lower strategic sensitivity, lower leadership unity and lower resource fluidity). Furthermore, firm age has also been studied in the context of strategic agility. For example, Doz (2020, p. 3) asserted that "*natural evolution leads to growing strategic rigidity as a company ages"* and Reed (2021b) performed a study with 30 firms from multiple industries located in the Space Coast of Florida, and stated that strategic agility declines as firms get older and that firms should use strategic agility before they lose it (or maintain it through exercise and training). Grounded in the former literature, we understand that:

#### Proposition 1:

Firm age is an influential element in strategic agility.

Firm size is also a well-known variable in the literature because it is an indicator of potential firm rigidity or flexibility. These studies are relevant insofar as flexibility is a requirement for achieving strategic agility (Roth, 1996; Weber & Tarba, 2014). According to Hannan and Freeman (1984), “the level of structural inertia increases with size for each class of organisation” (p. 158). This same approach is maintained nowadays through studies such as the one carried out by Corsi et al. (2019), who also explained that organisational inertia (understood as the force that slows down organisational change) increases significantly with firm size. They grounded their research on the assumption that large firms are associated with higher levels of organisational rigidity, while small firms are associated with flexibility. This position has been widely supported or extended in the literature to date. For example, van der Weerdt et al. (2006) proved that firm size had a negative impact on business flexibility (at operational, structural, and strategic levels). Verdú‐Jover et al. (2006) went further and analysed 417 European companies (large and small) to ascertain how their size affected their responses to changes in the environment; that is, their flexibility. Their findings showed that small firms were able to process information faster, although they found that large firms were better able to adapt to sudden changes in the environment. The reason behind this contradiction lay in the financial flexibility that large firms generally enjoy. Thus, although small firms are more flexible in nature, on many occasions they are not capable of making the necessary changes required to adapt due to the financial restrictions they suffer, while large firms, although more rigid in nature, have the necessary financial resources to implement the required changes. More recently, Haneberg (2021) explained that, in line with the literature, smaller firms adapt more easily to changes in the environment because they are naturally more flexible. Based on this approach, they analysed how SMEs were better able to adapt to unexpected crises (such as the COVID-19 crisis) than large firms. All this rationale suggests that firm size has a negative association with strategic agility.

In the strategic agility realm, some research has been carried out on firm size. For example, Oyedijo (2012) examined the relationship between strategic agility and competitive performance and suggested studying whether strategic agility is related to an organisation’s size and other attributes. In the same line, Bui et al. (2019) studied the impact of firm size on strategic renewal performance, which they associate with characteristics of strategic agility. Reed (2021b) also investigated strategic agility and its relationships with firm size and found that strategic agility did not decrease as firms grow larger.

Grounded in the above literature, we understand that:

#### Proposition 2:

Firm size is an influential element in strategic agility.

There are many kinds of strategic resources and capabilities common to businesses, such as technology, product development, production process, manufacturing or logistics (Desarbo et al., 2005) and firms need to use and develop new capabilities in order to benefit from the opportunities that arise from the external environment (Achtenhagen et al., 2013; Teece et al., 1997).

Authors have researched different resources and capabilities and their relation to strategic agility, such as human resources (Pina e Cunha et al., 2020), manufacturing procedures (Ofoegbu & Akanbi, 2012), and operations (Shin et al., 2015). However, the most studied capability as a potential source of strategic agility seems to be information technologies (IT), which has been reviewed in depth since the early 2000s. For example, Weill et al. (2002) asserted that senior executives make few choices that are more critical than deciding which IT investments will be needed for future strategic agility, and Ekman and Angwin (2007) studied 145 companies to gauge to what extent information systems and information technology (IS/IT) acted as an antecedent for strategic agility. They found that IS/IT was an important enabler for organisations belonging to a high-turbulence industry. Kappelman et al. (2014) underscored that cloud computing (referring to IT infrastructure capability) was one of the most important investments undertaken by organisations and used to develop strategic agility.

As with capabilities, resources can be of many types (Wernerfelt & Montgomery, 1988) and technology is one of the types of resources that most studies review. For example, Clauss (2017) asserted that new technologies are required to take into account opportunities (e.g., new product offering requiring a production technology, new revenue models requiring technical systems for paying).

In relation to strategic agility, the latest studies review how ‘new capabilities’ and ‘new technologies’ influence strategic agility. Clauss et al. (2019) considered these two elements as constructs where capabilities referred to employees receiving training to develop new competences, and employees having up-to-date knowledge and competences permanently assessed so as to adapt to changing market requirements, while technologies referred to firms’ up-to-date technical resources and innovative technical equipment to extend product and service portfolio.

Grounded in the previous literature, we understand that:

#### Proposition 3:

A firm´s capabilities and technologies are influential elements in strategic agility.

#### A firm’s business orientation associated with strategic agility

Despite internationalisation not being a common antecedent in the strategic agility literature, we find studies which highlight that strategic agility and firm internationalisation are two closely related elements (Demir et al., 2021; Shams et al., 2021). Furthermore, we found specific literature that explains how firms exposed to international markets are expected to learn and develop new capabilities; for example, new capabilities in innovation (Almodóvar & Nguyen, 2022; Almodóvar et al., 2014; Salomon & Jin, 2010; Salomon & Shaver, 2005). We might therefore expect international exposure not only to develop innovative skills but also to trigger new capabilities (for example, the meta-capability of strategic agility) to manage the diverse and rapid-changing international environment.

##### Proposition 4:

Firm internationalisation is an influential element in strategic agility.

We observe a recent increase in business model research in academia (Clauss et al., 2019) with one of the lines of research being how firms function, create and capture value (Spieth et al., 2014). There seems to be a consensus that business models consist of three dimensions: (a) value proposition, (b) value creation, and (c) value capture.(Clauss, 2017; Spieth & Schneider, 2016; Teece, 2010). Value proposition refers to how a product/service is composed, what role a firm has in production and delivery, what channels it uses and who is offered the company’s product/service (Morris et al., 2005); value creation refers to how value is created both at the firm and the external level (taking into account customers and suppliers) (Clauss, 2017); and the value capture dimension refers to how a firm makes money, considering new cost and revenue-related decisions, such as margins, quality and prices (Osterwalder & Pigneur, 2010). The ‘value capture’ dimension has been linked to strategic agility as it allows a firm to respond to changes in the environment through new revenue models and cost structures (Clauss et al., 2019).

Clauss et al. (2019) performed a study on 432 German firms in the electronics industry and reviewed the mediating role of ‘value capture’ in the relationship between strategic agility and firm performance. They considered ‘value capture’ as the combination of (a) new cost structures, and (b) new revenue models, (as Clauss (2017), who first used these combinations to create the construct). Contrary to their expectations, they found a negative effect between ‘value capture’ and firm performance. They therefore conducted additional semi-structured interviews to explore the relationship further. These interviews provided two key insights: first, that ‘value capture’ requires mutual adjustment from other parts in the system, and second that local optimisation problems can occur (e.g., local optimisation problems occurring due to different skillsets / required knowledge).

We can, therefore, expect ‘value capture’ (as a combination of new cost structures and revenue models) to have an effect on strategic agility. Specifically,

##### Proposition 5:

A firm's model for capturing value is an influential element in strategic agility.

#### A firm’s business environment associated with strategic agility

Turbulence in the environment is a relevant aspect because the purpose of strategic management is not to achieve certainty but to prepare the firm to face and survive uncertain environments (Von Oetinger, 2004). Turbulences can thus be caused by a variety of factors. Arifiani et al. (2021) pointed out that the most important sources of turbulence are the emergence of new technologies, changing market demands, increased intensity of competition (e.g., due to the entry of new competitors), and the appearance of new regulations. Complementing this approach, Rego et al. (2022) explained that the emergence of new technologies and changing market demands are two highly interconnected and mutually interdependent sources of environmental turbulence.

Regarding the connection between facing a turbulent environment and the need to activate protocols that lead to strategic agility, we consider that the appearance of innovations in the market brings together the effects of the above-mentioned two interconnected sources. In this line, we find some preliminary works –such as the research of Vagnoni and Khoddami (2016)– who explained that, among the various elements that cause a turbulent environment, the appearance of innovations in the market was highly relevant. The authors discussed that, in response to this situation, managers needed to implement alertness protocols to speed up the strategic changes required to respond to this threat. Another source of turbulence is the entry of new competitors into the industry, and this is a well-known threat that diminishes the attractiveness of industries (Porter, 2008). In fact, Ahmed et al. (1996) explained that, in the face of competitive pressures, firms need to develop rapid and quality responses and to increase their organisational flexibility in order to protect themselves against this new threat. Finally, Arifiani et al. (2021) stated that firms need to improve their strategic planning capabilities, and to be more creative and flexible in order to adapt to possible disruptive changes, such as new regulations. Summarizing these approaches, and in a more general manner, Pawłowski (2021) explained that there is a key relationship between unexpected and rapid changes in the environment (turbulent environments) and pointed to the need to increase the firm's flexibility in order to adapt and take advantage of new opportunities. In light of this area of work, we observe that turbulent environments negatively impact firms' activities and performance, such that enhancing strategic business agility is, therefore, a necessity (Fallmyr & Bygstad, 2014; Joiner, 2019; Lee & Wang, 2013).

In the strategic agility research line, several authors have analysed turbulent environments as an antecedent for agility (Reed, 2021b; Vazquez-Bustelo et al., 2007; Weber & Tarba, 2014). In this line, Afuah and Tucci (2003) asserted that fast-changing environments require being agile in perceiving and responding to changes in the environment to create innovations. van Oosterhout et al. (2006) studied changes in the environment (e.g., social/legal, competitive environment, customer needs) as factors required for agility. More recently, Ilmudeen (2021) explained that firms need to become more agile in order to be able to respond to turbulent environments. He therefore interviewed 254 senior executives from Chinese firms, and found that in turbulent environments, firms need to improve their dynamic capabilities facilitated by information technologies if they are to become more agile. Grounded in this literature, we understand that:

##### Proposition 6:

A firm's turbulent environment is an influential element in strategic agility.

## Methodology

After reviewing how academia explores the topic of strategic agility and the potential factors that are likely to interact with it, we built a survey that questioned respondents on the different factors related to strategic agility and the variable itself.

We study different cases and assess different potential configurations of factors for strategic agility, and therefore use QCA to explore the data. QCA has been used in the literature to assess different factors, in our case, internationalisation (Ciravegna et al., 2018; Fainschmidt et al., 2020; Verbeke et al., 2019), firm size (Greckhamer et al., 2007), and firm age (Ho et al., 2016), although there seem to be no studies addressing strategic agility.

In order to study the data, we use specific software (fsQCA 3.0) that helps identify whether each of the factors is required to reach the solution and which combinations of factors are sufficient to be a configuration that leads to strategic agility. The data required for the necessity and sufficiency analysis came from two sources: the survey and Orbis. This allowed us to ensure consistency in data and to obtain additional information (e.g., financial information). In order to use QCA, data had to be calibrated into the range [0–1], and we then analysed the results (different combinations of factors to show the alternative paths to strategic agility).

Finally, we interviewed one company that is representative of each alternative path so as to provide a tangible example of how specific companies operate and how they exhibit strategic agility.

### Data gathering

We prepared a survey with 40 questions that included both open and Likert scale questions. The survey was framed to the respondents as "Survey on company variables and competitions for organisations in Spain", and questions were grouped into eight different blocks: i) general company information, ii) financials, iii) internationalisation, iv) revenue models, v) cost structures, vi) strategy, vii) capabilities/technologies, and viii) environment/competition, such that respondents would not know the key variables being tested in our study.

We sent the survey to 71^1^ companies in Spain, specifically targeted to founding members of the companies or senior leadership (e.g., C-level executives or management committee members). Out of the surveys sent, we obtained 60 responses (response rate of 76%) from December 2021 to January 2022. We carried out telephone follow-ups to corroborate the accuracy and veracity of the data. Data from the survey were subsequently enriched and double-checked with company data from Orbis; namely sector of activity, operating revenue, net income, number of employees and date of incorporation. Finally, some firms were studied in more detail by reviewing media reports and undertaking semi-structured interviews.

Out of the 60 responses, we considered only a subset for the analysis, leaving out companies with one employee and those from a sector of activity other than services (e.g., textiles, industrial, metals, wholesale, computer software), leaving a final number of 40 respondents (Table 1).

### QCA and factors related to high levels of strategic agility

QCA is a powerful tool for analysing causal complexity; that is, when i) an event occurs given a combination of causal factors, ii) different combinations of causal factors can lead to the same outcome, and iii) causal factors may work differently in different cases (i.e., depending on combinations with other factors) (Greckhamer et al., 2007).

There are different types of QCA analyses. We use a fuzzy-set QCA (FsQCA) approach because it allows variables to obtain all the values within the range [0–1] (Pappas & Woodside, 2021) and because it has received increased attention recently (Fiss, 2011; Ordanini et al., 2014; Pappas et al., 2016; Woodside, 2014).

FsQCA aims to identify necessary and sufficient conditions and the relationships that associate with the outcome of interest (Douglas et al., 2020). However, in order to avoid "researcher degrees of freedom" (Gelman & Loken, 2014), antecedent conditions selected for the configurational model must be supported by extant theory or be accompanied by plausible propositions for new theory (Greckhamer et al., 2018). In other words, the model should only include those antecedent conditions that prior theory, informed reasoning or prior surprising findings suggests are likely to interact with each other (Douglas et al., 2020).

We, therefore, study the relations between the following factors (antecedents) and strategic agility:

1. Firm age: this is a factor frequently studied in the literature (Reed, 2021b) and is commonly measured as the number of years since a firm’s foundation (Autio et al., 2000), which is how we measure this factor.
2. Firm size: also a common factor in studies and which is measured in several ways, such as number of employees, annual revenue or assets (Reed, 2021b). We measure the factor using the number of employees since there is less sensitivity to its reporting by some firms.
3. Internationalisation: we measure internationalisation by asking survey respondents whether their firm operates only in Spain or whether it has any kind of activity overseas (exports or subsidiaries). Given that there are hardly any previous studies on internationalisation and strategic agility, we decided to generalise and to take into account any kind of international exposure.
4. Turbulent environment: we measure environmental turbulence by asking survey respondents whether they have experienced either new entrants, innovations, or new regulations that altered the environment. Although some studies, such as Reed (2021b), also take into account the customer dimension, we decided to remove this question in the questionnaire following the latest studies by Arifiani et al. (2021) and Rego et al. (2022), who pointed out that the most important sources for turbulence are innovations, new competition and new regulation, and that innovations and changing market demands are two highly interconnected and mutually interdependent sources of environmental turbulence.
5. Capabilities or technologies: following Clauss et al. (2019), we included the same questions in the survey so as to understand whether a firm invests in new capabilities and technologies.
6. Value capture: following Clauss et al. (2019), we included the same questions in the survey so as to understand whether a firm develops new revenue models and seeks cost-saving opportunities

Finally, following Clauss et al. (2019), we measure strategic agility by asking survey respondents nine questions, three for each of the components (strategic sensitivity, leadership unity, and resource fluidity).^2^

### Data calibration

Data must be "calibrated" to enable Boolean analysis. That is, fsQCA requires each of the factors to be within the range [0–1], and the purpose of calibration is to choose threshold data scores that the researcher considers will reflect that the respondent is either "fully in" the set, or "fully out" in terms of “membership”. Between these threshold scores, there is ambiguity as to whether the score is in or out of the set, and a point of maximum ambiguity must be set (Greckhamer et al., 2018).

Calibration for each variable depends on whether variables are binary, multi-value, or continuous. For binary variables (crisp factors), researchers establish 1 as representing "fully in" and 0 as representing "fully out". For fuzzy factors such as multi-value sets (e.g., a 5-point Likert scale) or continuous variables (e.g., revenue), researchers must apply theoretical and context knowledge to identify the most appropriate threshold scores that imply full membership, full non-membership and the point of maximum ambiguity (Greckhamer et al., 2018; Hannan, 2010; Ragin, 2008; Verkuilen, 2005). We calibrate crisp factors with 1 or 0 according to the criteria in Table 2.

**Table 1:** Characteristics of firms in the study

**Sector of activity**	Health Social Services, Business Services, Property Services, Financial Services
**Employees**	2 – 123 k
**Operating revenue (USD)**	$1,7 k – $6b
**Net Income (USD)**	-$1,3b – $2,5b
**Date of incorporation**	1870 – 2020

**Table 2:** Calibration of the crisp factors

**Factor**	**Name of the factor in the model**	**Source**	**Full membership (Calibration to 1)**	**Full non-membership** **(Calibration to 0)**
Internationalisation	International	Survey (one question)	Companies that operate in more than one country	Companies that operate only in one country
Turbulent Environment (New entrants, innovation, or regulation)	EntInnReg	Survey (three questions)	Companies that have responded positively to either of the three questions	Companies that have responded negatively to all three questions
Size	SmallSize	Survey (one question) & Orbis	Companies that have less than 250 employees	Companies that have 250 employees or more

For fuzzy factors, we use percentiles 99, 50 and 1 to find which values in our dataset correspond to the thresholds for full membership, point of maximum ambiguity, and full non-membership (see Table 3). With these thresholds per factor, we calibrate the data (Ragin & Davey, 2016).

**Table 3:** Calibration of the fuzzy factors

**Factor**	**Name of the factor in the model**	**Source**	**Full membership**	**Point of maximum ambiguity**	**Full non-membership**
Firm Age	YoungAge	Orbis (current year minus year of foundation)	Value according to the percentile 1 of the data (1.39 years)	Value according to the percentile 50 of the data (14.5 years)	Value according to the percentile 99 of the data (189.01 years)
Capabilities or technologies	CapOrTech	Survey (three questions on 'New capabilities' and three on 'New technologies' which were then averaged)	Value according to the percentile 99 of the data (4.67)	Value according to the percentile 50 of the data (3.83)	Value according to the percentile 1 of the data (2.80)
New cost structures / revenue models	ValueCapture	Survey (three questions on 'new revenue models' and three on 'new cost structures' which were then averaged)	Value according to the percentile 99 of the data (5)	Value according to the percentile 50 of the data (3.58)	Value according to the percentile 1 of the data (1.2)
Strategic agility	STRATEGICAGILITY	Survey (nine questions on strategic agility (strategic sensitivity, leadership unity and resource fluidity) which were then averaged)	Value according to the percentile 99 of the data (4.89)	Value according to the percentile 50 of the data (3.72)	Value according to the percentile 1 of the data (2.08)

### Setting the consistency threshold and conducting "necessity analysis"

Table 4 shows that there are no 'necessary conditions' for Strategic Agility to occur, as we have no condition that exhibits a consistency above 0.90 with non-trivial coverage (Schneider, 2018; Schneider & Wagemann, 2012).

**Table 4:** Analysis of Necessary conditions for the outcome variable STRATEGICAGILITY

**Conditions tested**	**Consistency**	**Coverage**
	STRATEGICAGILITY	
SmallSize	0.482	0.517
~SmallSize	0.518	0.556
International	0.649	0.557
~International	0.351	0.503
EntInnReg	0.806	0.577
~EntInnReg	0.194	0.416
YoungAge	0.809	0.716
~YoungAge	0.552	0.751
CapOrTech	0.786	0.823
~CapOrTech	0.573	0.630
ValueCapture	0.791	0.827
~ValueCapture	0.600	0.661

Once data is calibrated, we set the "consistency threshold" at 0.90, which is above the acceptable level of dissimilarity of within-case relationships between the outcome (Rihoux & Ragin, 2009) and above the ‘best-practice’ threshold of 0.80, which provides greater homogeneity within configurations (Douglas et al., 2020).

Table 4 shows the truth table after calibration and setting the consistency threshold. Additional to the consistency threshold, we also take into account the proportional reduction in inconsistency (PRI) threshold, which is an important measure to avoid simultaneous subset relations of configurations in the outcome and its negation. We set this threshold at 0.75 to eliminate configurations with low PRI scores that indicate inconsistency (Greckhamer et al., 2018) and at a level classified as preferable in the literature (Frambach et al., 2016). Table 5 in bold font shows those cases with a PRI consistency above the threshold, and which are, therefore, the ones used by fsQCA to identify solutions.

**Table 5:** Truth table in fsQCA, where cases in bold exceed the PRI consistency threshold

SmallSize	International	EntInnReg	YoungAge	CapOrTech	ValueCapture	STRATEGIC AGILITY	Raw consist	PRI consist	SYM consist
**1**	**0**	**1**	**1**	**1**	**1**	**1**	**1**	**1**	**1**
**1**	**0**	**0**	**1**	**1**	**1**	**1**	**0.992**	**0.988**	**0.988**
**0**	**1**	**1**	**1**	**1**	**1**	**1**	**0.958**	**0.914**	**0.914**
**1**	**1**	**1**	**1**	**1**	**1**	**1**	**0.956**	**0.875**	**0.875**
**1**	**1**	**1**	**1**	**1**	**0**	**1**	**0.942**	**0.871**	**0.871**
**0**	**1**	**1**	**0**	**1**	**1**	**1**	**0.930**	**0.853**	**0.870**
**1**	**1**	**1**	**0**	**0**	**0**	**1**	**0.939**	**0.838**	**0.838**
**1**	**0**	**0**	**1**	**1**	**0**	**0**	**0.876**	**0.811**	**0.811**
**0**	**1**	**1**	**1**	**0**	**1**	**1**	**0.913**	**0.787**	**0.787**
1	0	1	1	1	0	1	0.926	0.592	0.879
0	1	1	0	0	1	0	0.831	0.524	0.524
1	1	1	1	0	0	0	0.804	0.431	0.431
1	1	0	0	0	1	0	0.893	0.348	0.348
0	1	1	0	0	0	0	0.828	0.253	0.274
0	1	1	0	1	0	0	0.764	0.243	0.246
0	1	1	1	0	0	0	0.783	0.235	0.235
1	0	1	1	0	1	0	0.715	0.230	0.230
0	0	1	1	0	1	0	0.781	0.178	1
1	0	0	1	0	0	0	0.413	0.110	0.110
0	0	1	0	0	1	0	0.897	0	0
1	0	1	0	1	1	0	0.872	0	0
0	1	0	0	0	0	0	0.857	0	0
0	0	1	1	1	1	0	0.813	0	0
1	1	0	0	0	0	0	0.758	0	0
1	1	0	1	0	0	0	0.682	0	0
0	0	0	0	0	0	0	0.623	0	0
1	1	0	1	0	1	0	0.607	0	0
1	0	0	0	0	0	0	0.402	0	0

## Results: obtaining the configurations/solutions and illustrations of real cases

FsQCA computes three solutions (a combination of configurations supported by a high number of cases, with a consistent rule of "the combination leads to the outcome") (Pappas & Woodside, 2021). The complex solution presents all possible combinations of conditions, including combinations not observed in the data. This solution is further simplified into the parsimonious and intermediate solutions.

The parsimonious solution is a simplification of the complex solution, which includes "core conditions" –in other words, those that cannot be left out from any solution (Fiss, 2011). The intermediate solution is obtained by performing counterfactual analysis on the complex and parsimonious solutions, including only theoretically plausible counterfactuals (Liu et al., 2017; Ragin, 2008). We combine the parsimonious and intermediate solutions to offer a more detailed and aggregated view of the findings (Fiss, 2011). This combination of the two solutions led to five different configurations or recipes that firms use to achieve strategic agility (see Table 6, where we have visually represented the different configurations). It should be noted that our model has a consistency of 0.93, which is considered useful and can serve to advance theory (Woodside, 2017).

**Table 6:**
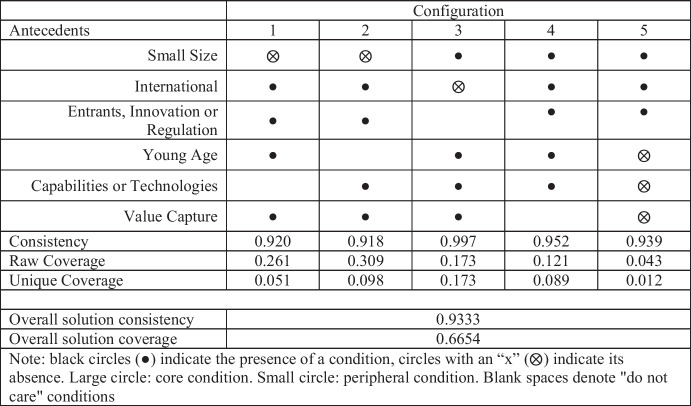
FsQCA findings for Strategic Agility

### Configurations 1 and 2:

*Larger international firms competing in turbulent environments that actively invest in new revenue-increasing and cost-decreasing opportunities.*

Firms in these configurations are characterised by having > 250 employees, being international, and competing in turbulent environments (i.e., firms have faced entrants that altered the status quo of incumbents, or innovation/ regulation has changed the 'rules of the game'). Additionally, firms in these configurations develop new revenue opportunities (e.g., complementing/replacing one-time transaction revenues with long-term recurring revenue models) and actively seek cost reduction opportunities. Configurations 1 and 2 are very similar, with 1 referring to firms that are young, and configuration 2 to those firms that additionally make an effort to keep up to date in technologies and capabilities (e.g., employees receive constant training, competencies reflect changing market requirements, technical employment is up-to-date).

DeliveryCo^3^ is one of the companies representative of configuration 1. This company was established in 2015 and grew quickly to have over 4,000 employees as of 2022. The company competes in the delivery business, both for takeaway food and as a general courier service, but has recently expanded into other businesses, most notably quick commerce, and small home repairs. DeliveryCo was born in Spain, but quickly internationalised and as of 2022 is operating in 22 European countries. The environment in which DeliveryCo competes is very turbulent, with new disruptive entrants (e.g., companies backed by private equity that have both a well-crafted plan and resources to compete in the industry), innovation (e.g., self-driving and autonomous vehicles that could displace traditional delivery services), and regulation (e.g., country and EU-level legislation that govern how delivery companies can operate). DeliveryCo has been very active in developing new revenue models and cost structures. The company frequently develops and tests new ideas in the markets (e.g., high-quality food delivery, mobility scooters), launched a recurring revenue model (monthly flat-fee that allows customers to avoid delivery fees), and actively seeks price differentiation taking into account their costs (e.g., delivery fees for restaurants vary according to time and distance from the client). Finally, DeliveryCo exhibits all the components of strategic agility i) strategic sensitivity, ii) leadership unity, and iii) resource fluidity as the company is very sensitive to external changes and quickly anticipates/reacts to these changes, management is able to make bold and fast strategic decisions collaboratively, and resources are fluidly reallocated as required.

BankCo is one of the companies that represent configuration 2. It has been competing in the banking industry for over 100 years and has over 100,000 employees worldwide, as it operates in several countries. The environment where BankCo competes faces both new entrants (e.g., fintech companies), innovation (e.g., decentralised finance, blockchain), and regulation (e.g., General Data Protection Regulation in Europe). BankCo has been very active in developing capabilities and technologies and in developing new revenue models and cost structures. As regards the former, BankCo ensures that its employees receive frequent training to develop new competences, and the company considers that its employees have more up-to-date knowledge than its competitors. Likewise, BankCo invests in keeping the company’s technical resources up to date and the company considers its technical equipment to be very innovative. As regards revenue models and cost structures, BankCo is frequently developing new revenue opportunities (e.g., innovative credit cards) and is actively seeking cost reduction opportunities (e.g., closing branches, resizing departments). As with DeliveryCo, BankCo exhibits all the components of strategic agility.

### Configuration 3:

*Young and small national firms that actively invest in revenue increasing and cost decreasing opportunities*

Firms in this configuration are characterised by their lack of international presence, being small and young, regardless of how stable or turbulent the environment is. However, these firms actively seek opportunities to develop new revenue and reduce costs.

ConsultingCo is an example of these companies. ConsultingCo was created in 2018 and operates with five employees only in the Spanish market. The company performs consulting projects across several domains (e.g., commercial assessments and introduction of software applications in the Spanish market,). The company actively develops new revenue opportunities by seeking attractive models for its customers. For example, it started billing clients on the number of hours dedicated to a project and then changed to commission-based work, where ConsultingCo earns according to variable metrics and the success of the project. Likewise, the company is actively working on making its cost structure as variable as possible (e.g., by moving from a leased office to working in a shared business centre, or by having a reduced cost base where only the partners are on the payroll of the company and where all additional consultants required for each project are hired on a project basis).

ConsultingCo actively invests in capabilities (providing its consultants with several training activities depending on the projects they are involved in) and technologies (providing necessary technology to report progress on the project and to show the client what additional areas are pending). ConsultingCo exhibits all the components of strategic agility, where they pay special attention to strategic sensitivity (by actively looking at what competitors are offering and by capturing client feedback), and resource fluidity (by adjusting resources to ensure the talent staffed in each project matches what the client requires).

### Configuration 4:

*Small and young international firms that compete in turbulent environments.*

This configuration is comprised of firms that are relatively young and small but which have, nevertheless, already sought to internationalise. Likewise, these companies compete in turbulent environments.

HRCo exemplifies this configuration. It is a very new company (founded in 2020) that was born with the aim of enabling people to access their earned salary at any point in the month (i.e., on the fifth day of a given month, an employee can access five days’ worth of salary). As of late 2021, the company had 30 employees and operated in three countries: Spain, Italy, and Colombia. HRCo competes in a turbulent environment, mostly driven by the appearance of new entrants in their industry (e.g., a large international fintech has recently offered a “Pay by Day” feature that directly competes against HRCo). As regards strategic agility, HRCo exhibits all of the components, although the company’s stronger perception of leadership unity is the component that most stands out (i.e., HRCo is particularly strong at having top management making bold and fast strategic decisions collaboratively).

### Configuration 5:

*Older small international firms that compete in turbulent environments.*

While configurations 1, 2, 3, and 4 exhibit high raw coverage values, configuration 5 has only 0.043. However, this does not mean that it is not important (Rubinson et al., 2019). Although it is the least common path for strategic agility for firms, configuration 5 shows an alternative for some firms.

Firms in this configuration are those that are older, small, and that are international companies who compete in turbulent environments. However, these companies do not make a particular effort to keep up to date in technologies and capabilities. Nor do they develop new revenue-increasing or cost decreasing opportunities.

CloudCo exemplifies this configuration. It is a company established in 2001 that competes in the industry of software for contact centres (i.e., as contact centres unify different channels, such as telephone, WhatsApp, or chat, this software helps unify the different alternatives). As of late 2021, CloudCo has a little over ten employees, but operates in several countries, particularly in Latin America, such as Mexico or Colombia. CloudCo competes in a very turbulent environment, where it has to cope with new entrants, frequent innovation and regulatory changes. New entrants frequently appear, particularly from countries that have a low-cost high-skilled workforce, such as India. CloudCo actively monitors 150 competitors, and every month they have new additions. Innovation also poses a threat to their business, and the company is particularly concerned about artificial intelligence. They face a risk of very intelligent systems being able to independently attend to customers (i.e., a machine-centred WhatsApp that is able to have a natural dialogue with a user calling a contact centre, without noticing that the counterpart is not human). Regulation is the biggest threat, as perceived by CloudCo. Every country has a different regulation that they have to keep up to date with, and data protection laws are constantly appearing. As for strategic agility, CloudCo exhibits all of its components, with the company having a stronger perception of leadership unity as the most important component.

## Conclusions

The objective of this paper is to assess what factors are related to strategic agility. As practical knowledge on the topic suggested there might be several alternatives for firms to achieve strategic agility, we used QCA, a technique that allows different combinations of factors to be identified that lead to the outcome. We surveyed 40 companies in the services sector in Spain in an effort to understand their specificities in terms of firm size, firm age, internationalisation, environmental turbulence, investment in capabilities and technologies as well as revenue-increasing and cost-cutting mechanisms. Finally, we concluded the study with semi-structured interviews for one company representative of each configuration. This paper makes important contributions to the literature. First, it shows that there is no single factor that is necessary for firms to accomplish strategic agility. Second, we show there are different combinations of factors that lead firms to display strategic agility. This means that different types of firms can reach strategic agility in alternative ways. Third, we present real cases that illustrate each configuration with a real example.

### Theoretical and managerial implications

Through our research, we seek to shed light on an underexplored phenomenon of great relevance to academia; namely, strategic agility. However, the main theoretical contribution is that we provide a better understanding of the phenomenon at hand, with our results indicating that there are various firm configurations which are strongly linked to high levels of strategic agility. The results we obtained display high consistency, suggesting that the model is useful and can serve for theory advancement (Woodside, 2017). Specifically, we unveil five paths or configurations that favour firms to achieve strategic agility.

- Configurations 1 and 2 are the most common and refer to larger international firms that compete in turbulent environments. These firms deal with new entrants, innovations and/or regulations and make several efforts to identify ways to increase revenues and reduce costs. Configuration 1 refers to firms that are young (i.e., established in the last couple of years), and configuration 2 refers to firms that invest in capabilities or technologies, in addition to making efforts in terms of revenue increasing and cost decreasing initiatives.
- Configuration 3 refers to young, small firms with no international presence. Regardless of whether the environment is calm or turbulent, firms in this path provide their employees with the up-to-date capabilities and innovative technologies required to perform their job. Likewise, firms actively develop new revenue opportunities and seek cost-saving opportunities.
- Configuration 4 is the path followed by young and small firms that have an international vocation and that compete in turbulent environments.
- Configuration 5 is the least common and refers to older firms, but who still have a small number of employees and who have decided to compete in the international arena and face a turbulent environment.

From a managerial perspective, a desire for practical knowledge on the topic provided the incentive for this study and thus sought to identify practical implications beyond the purely academic. In particular, identifying the paths or configurations is interesting not only for academics but also for practitioners, since they can compare the situation in their current firms with that of the real cases provided in each of the configurations This will help them identify the gaps and will offer an indication of how they can reach strategic agility in their companies.

## Limitations and future research

Our study provides important contributions but is not free from limitations. We use a sample of 40 respondents from a specific sector and country, which implies that the sample may not be representative of a wider population and that conclusions cannot be extrapolated in a general sense.

Our sample size is appropriate, since QCA is a reasonable choice of method for sample sizes of 12 or more, with the minimum sample size depending on the number of causal conditions in the model (e.g., seven causal conditions requiring a sample of about 30) (Marx, 2006). However, having access to a larger sample may standardise the results provided by each company (provided that more responses per company are averaged).

QCA requires identifying the potential factors to be studied beforehand. Following Greckhamer et al. (2018), we selected the antecedent conditions for the model through an exhaustive review of the literature and explained propositions on why we think these conditions need to be analysed.

Additionally, QCA requires data to be ‘calibrated’ in order to enable Boolean analysis. The purpose of this is to choose threshold data that the researcher can use to judge whether a response is ‘fully in’ or ‘fully out’ of the set. The number of potential calibrations is virtually endless. We chose to use data frequency distribution and to use percentiles 1 and 99 to determine cut-off points, and we stated and applied this rationale across conditions to avoid distortion of results (Douglas et al., 2020).

Finally, our model showed a coverage of 0.66, which means that approximately two-thirds of the outcome of interest (strategic agility) is explained by the configurations. This is a comparable measure to the R-square reported on regression-based methods (Woodside, 2013). While the coverage obtained is high, there is still a part of the outcome of interest that is not explained. We, therefore, acknowledge that we do not examine all possible antecedents of strategic agility, such that future research lines might focus on additional factors that are worth studying. For example, there is emerging evidence that there might be some relationship between entrepreneurship and strategic agility, with (e.g., (Kwon et al., 2018) providing evidence from South Korea). New studies might thus assess entrepreneurship and entrepreneurial intentions as the first step in the process (Palmer et al., 2019), considering the tensions between academic identity and entrepreneur identity (Shi et al., 2021).

Strategic agility is an emerging topic (de Diego & Almodovar, 2021) and, therefore, several research questions can be pursued to explore the topic in greater depth. For example, researchers may wish to perform more in-depth studies on each of the factors that lead to strategic agility (e.g., firm size, firm age, internationalisation). More explicitly, the antecedent “internationalisation” was not analysed before in this context and it has proved its relevance. Future research might therefore further investigate more specific aspects of firms' internationalisation in order to discern its effects on strategic agility.

Likewise, it would be interesting to identify differences in factors leading to strategic agility when comparing sectors and to assess the differences between the services sector and, for example, the manufacturing sector. Finally, there might be opportunities to analyse cross-cultural differences between countries by performing the same study on companies in different geographical areas and by comparing those results to the ones presented here for Spanish firms.

## Data Availability

The raw/processed data necessary to reproduce our results and conclusions cannot be shared to ensure the protection of the privacy and confidentiality of the respondents.

## Appendix A Questionnaire used for data calibration

**Table:**

**QUESTION**	**TYPE**
**Section on internationalisation**	
How many countries does your company operate in (taking into account: (1) countries where you have subsidiaries plus (2) countries where you export)?	Open

**Section on revenue models**	
We recently developed new revenue opportunities (e.g., additional sales, cross-selling)	5-point Likert
We increasingly offer integrated services (e.g., sale of product plus a maintenance contract) in order to realise long-term financial gains	5-point Likert
We recently complemented or replaced one-time transaction revenues (e.g., single sales) with long-term recurring revenue models (e.g., leasing)	5-point Likert

**Section on cost structures**	
We actively seek for opportunities to save manufacturing costs	5-point Likert
Our production costs are constantly examined and if necessary, amended according to market prices	5-point Likert
We utilise opportunities, which arise through price differentiation	5-point Likert

**Section on strategy**	
We are very sensitive for external changes (regarding customers, competitors, technologies, etc.) and integrate these into strategic planning of our company	5-point Likert
We utilise different mechanisms to become aware of strategic developments early (i.e., we survey our clients, perform market studies, assess competitors, assist to conferences)	5-point Likert
Requirements for strategic adaptations are communicated fast and comprehensively through the organisation	5-point Likert
Our top management is able to make bold and fast strategic decisions	5-point Likert
Our management collaborates for strategic decisions	5-point Likert
Strategic decisions are collectively solved by our management without being bogged down in top-level “win-lose” politics	5-point Likert
We are able to reallocate and utilise capital resources fluidly	5-point Likert
Our people and their competencies are highly mobile within our organisation	5-point Likert
Our organisational structure allows for flexible redeployment of our resources	5-point Likert

**Section on capabilities / technologies**	
Our employees constantly receive trainings in order to develop new competences	5-point Likert
Relative to our direct competitors, our employees have very up-to-date knowledge and capabilities	5-point Likert
We permanently reflect which new competencies need to be established in order to adapt to changing market requirements	5-point Likert
We keep the technical resources of our company up-to-date	5-point Likert
Relative to our competitors, our technical equipment is very innovative	5-point Likert
We regularly utilise new technical opportunities in order to extend our product and service portfolio	5-point Likert

**Section on environment / competition**	
In the last three years, have there been new entrants to your industry with the ability of altering the status quo of the incumbents?	Dichotomous
In the last three years, has there been any innovation that has ‘changed the rules of the game’ significantly in your industry (e.g., electric vehicles in Automotive industry)?	Dichotomous
In the last three years, has there been any significant regulation/law that has ‘changed the rules of the game’ significantly in your industry (e.g., GDPR in Financial Services industry)	Dichotomous

## Footnotes

1 As standard practice in QCA studies –a case-based method– company selection does not correspond to a representative sample of the Spanish services sector in the traditional sense, but to a heterogeneous and relevant sample of the sub-sectors: Health Social Services, Business Services, Property Services, Financial Services.

2 In Appendix A, we include the exact wording of the questions linked to the variables of this research.

3 Firms have been disguised to protect confidential data.
